# Electrocardiographic Features in Patients with Neuromuscular Diseases: A Retrospective Analysis

**DOI:** 10.3390/diagnostics16213582

**Published:** 2026-10-30

**Authors:** Pietro Pugliatti, Francesco Catanzariti, Giancarlo Trimarchi, Pierangela Irrera, Gianluca Di Bella, Francesco Luzza

**Affiliations:** 1 Cardiology Unit, Department of Clinical and Experimental Medicine, University of Messina, 98122 Messina, Italy; francescocatanzariti96@gmail.com (F.C.); pierangela.irrera@unime.it (P.I.); gianluca.dibella@unime.it (G.D.B.); francesco.luzza@unime.it (F.L.); 2 Interdisciplinary Center for Health Sciences, Scuola Superiore Sant’Anna, 56127 Pisa, Italy; giancarlo.trimarchi18@gmail.com; 3 Fondazione Toscana G. Monasterio, Ospedale del Cuore “G. Pasquinucci”, 54100 Massa, Italy

**Keywords:** atrial arrhythmias, atrial myopathy, Becker Muscular Dystrophy (BMD), deep Q waves, Duchenne Muscular Dystrophy (DMD), electrocardiogram (ECG), Myotonic Dystrophy Type 1 (DM1), neuromuscular diseases (NMDs), P-wave voltage

## Abstract

**Background:** Neuromuscular diseases (NMDs) are a group of progressive hereditary disorders frequently associated with significant cardiac involvement, which can represent a primary cause of mortality. This study aims to characterize the electrocardiographic findings in patients with Duchenne Muscular Dystrophy (DMD), Becker Muscular Dystrophy (BMD), and Myotonic Dystrophy Type 1 (DM1). **Methods:** We conducted a retrospective analysis of 131 ECGs from 74 patients (28 DMD, 24 BMD, 22 DM1) followed in the Department of Clinical and Experimental Medicine of the University of Messina, Italy. None of the enrolled patients had clinically manifest heart failure. Electrocardiographic and echocardiographic data were collected, and each ECG finding was considered present if documented in at least one ECG during follow-up. In the DM1 group, an exploratory analysis of the 12-lead P-wave voltage sum (ΣP) and of its ratio to the QRS voltage sum (ΣQRS) was performed and compared with DMD and BMD patients and healthy controls. **Results:** In DMD patients, the most common findings were deep Q waves (57.1%), negative T waves in the lateral leads (39.3%), and complete and incomplete right bundle branch block (RBBB) (25.0% each). In BMD, incomplete RBBB (27.3%), U waves (27.3%) and left atrial enlargement (23.8%) were prevalent, and 3 patients (12.5%) had a pacemaker. In DM1, atrial fibrillation (18.2%), PR interval prolongation (38.9% of patients in sinus rhythm) and early repolarization (42.9%) were frequent. Mean ΣP and ΣP/ΣQRS values were lower in DM1 patients than in DMD and BMD patients and healthy controls. **Conclusions:** This study confirms many previously described ECG abnormalities in NMDs and highlights disease-specific patterns. The finding of deep Q waves in DMD may reflect inferolateral fibrosis. Notably, the reduced P-wave voltage in DM1 patients is a novel, hypothesis-generating observation that may reflect atrial myopathy; its value as a predictor of atrial arrhythmias requires prospective validation. These findings underscore the critical role of routine ECG surveillance in the management of patients with NMDs.

## 1. Introduction

Neuromuscular diseases (NMDs) constitute a heterogeneous group of progressive, inherited pathologies caused by mutations in various genes, leading to structural alterations and functional loss in muscle tissue [1,2,3,4]. These disorders vary in their clinical presentation, including the age of onset—ranging from infancy to adulthood—and the specific muscle groups affected. Involvement of the cardiac and respiratory muscles is common and represents a primary cause of mortality in many of these conditions [5,6].

Among the most common NMDs with childhood onset are Duchenne Muscular Dystrophy (DMD) and Becker Muscular Dystrophy (BMD), both X-linked recessive disorders resulting from mutations in the dystrophin gene. Dystrophin is a protein that is essential for muscle fiber integrity. Duchenne Muscular Dystrophy represents the more severe form and arises from frameshift mutations or large deletions that disrupt the reading frame, resulting in either absent or non-functional dystrophin. Becker Muscular Dystrophy is caused by in-frame mutations that permit the production of partially functional dystrophin, resulting in a comparatively milder phenotype [7,8,9,10].

The most frequent adult-onset muscular dystrophy is Myotonic Dystrophy Type 1 (DM1), also known as Steinert’s disease, an autosomal dominant disorder caused by a trinucleotide repeat expansion. This repeat expansion results in toxic RNA aggregates that interfere with RNA splicing factors and cause widespread disruptions in RNA processing and cellular function. Clinical severity in DM1 also correlates with repeat length: larger expansions are associated with earlier onset and more severe disease manifestations [11]. Moreover, in Steinert’s disease, abnormal splicing of *SCN5A* with subsequent cardiac sodium channel dysfunction has been identified as an important contributor to the genesis of arrhythmias in DM1 [12].

In patients affected by NMDs, the progressive nature of muscle deterioration significantly reduces quality of life; furthermore, these patients demonstrate heightened susceptibility to comorbid conditions, particularly pulmonary and cardiovascular diseases, and life expectancy is considerably reduced, mainly due to respiratory and cardiac failure [3,7].

Cardiac manifestations may include cardiomyopathy and disorders of the cardiac rhythm. When present, dilated cardiomyopathy is the most common, but hypertrophic cardiomyopathy has also been described in some cases. Arrhythmias are frequent in NMDs, and are represented by both atrial and/or ventricular arrhythmias, as well as by disorders of the atrioventricular and intraventricular conduction system. In many cases, arrhythmias appear to be the consequence of a clinically evident cardiomyopathy, but in many other cases, they can manifest in the absence of clinically evident heart disease and represent the first expression of cardiac involvement in these patients. Consequently, an accurate cardiac evaluation based on the search for possible ECG alterations is of fundamental importance for the early detection of cardiac involvement in these patients, and for timely intervention [13]. The aim of our study was to evaluate the presence of any electrocardiographic alterations and their incidence in patients affected by three major NMDs, in the absence of clinically manifest heart failure.

## 2. Materials and Methods

In this retrospective study, we evaluated the electrocardiographic and echocardiographic records of patients with muscular dystrophies from Sicily and Calabria who were followed at the clinical center for neuromuscular diseases at the Department of Clinical and Experimental Medicine of the University of Messina. ECGs were recorded between 2002 and 2021 (all but two between 2012 and 2021). Of the 75 patients in the study registry, one BMD patient without an available ECG was excluded, leaving 74 patients for analysis. None of the enrolled patients had a clinically manifest picture of heart failure nor were they undergoing cardiovascular drug treatment. The study was conducted in accordance with the Declaration of Helsinki, and the study protocol was registered by the Local Ethics Committee (Comitato Etico Locale, CEL) under protocol number 110-26, approval date: 12 October 2025.

The patients were divided into three groups:

- Group 1 (DMD): 28 male patients with Duchenne Muscular Dystrophy, aged 10 to 37 years at the first ECG.
- Group 2 (BMD): 24 male patients with Becker Muscular Dystrophy, aged 16 to 79 years at the first ECG.
- Group 3 (DM1): 22 patients with Myotonic Dystrophy Type 1 (12 males, 10 females), aged 26 to 76 years at the first ECG.

Baseline demographic and echocardiographic characteristics of the three patient groups are summarized in Table 1.

For each patient, all available ECGs (131 in total; median 2 per patient, range 1–3) and echocardiograms performed during the follow-up period were reviewed independently by two different operators, and the results were recorded in a spreadsheet. Each ECG finding was considered present if it was documented in at least one ECG during follow-up. P-wave and PR interval findings were assessed only on ECGs recorded in sinus rhythm, whereas QRS, ST-segment, T-wave and QT findings were assessed on all non-paced ECGs; the denominator of each percentage is reported in the text and tables. Interobserver agreement was not formally assessed.

Echocardiographic analysis included the assessment of cardiac chamber thicknesses and diameters, valve structure, and left ventricular ejection fraction (LVEF). Left ventricular and left atrial dilation were recorded as reported in the echocardiographic reports, and for each patient, the lowest LVEF recorded during follow-up was considered.

The ECG analysis evaluated the baseline rhythm (sinus, atrial fibrillation/flutter, paced), P-wave morphology and duration, PR interval, QRS morphology and duration, ST segment, T-wave morphology, the presence of U waves and QTc interval (calculated by QT interval and heart rate using Bazett’s formula).

Electrocardiographic abnormalities were defined according to current recommendations [14]. A prolonged PR interval (first-degree atrioventricular block) was defined as PR > 200 ms and a short PR interval as PR < 120 ms. Complete and incomplete right and left bundle branch blocks and left anterior/posterior fascicular blocks were defined according to the AHA/ACCF/HRS criteria [14]. A tall R wave in V1 was defined as an R wave greater than the S wave (R/S ratio > 1) in lead V1. Prominent (deep) Q waves were defined as Q waves deeper than 0.2 mV or exceeding 25% of the amplitude of the entire QRS complex, in the inferior (II, III, aVF) and/or lateral (I, aVL, V4–V6) leads. Early repolarization (ER) was defined as J-point elevation ≥ 0.1 mV (1 mm) in ≥2 contiguous inferior and/or lateral leads; ST-segment elevation confined to the right and mid precordial leads was reported separately. The QT interval was corrected for heart rate with Bazett’s formula and classified as normal or prolonged.

A specific analysis was conducted for patients with DM1. After observing a relatively frequent occurrence of low-amplitude P waves in this group, in each patient of the group we quantified the P-wave voltage by calculating the sum of the absolute P-wave voltages across all twelve leads (ΣP). To normalize for factors like body habitus, we also calculated the sum of the absolute values of all QRS waves in the twelve leads (ΣQRS) and computed the ratio ΣP/ΣQRS for each patient. Because this analysis was conceived after the initial review of the tracings, it should be considered exploratory (post hoc). Only subjects in sinus rhythm were included, i.e., patients with atrial fibrillation/flutter or with a pacemaker were excluded; the analysis therefore included 18 DM1 patients. The values obtained in DM1 patients were compared with those from DMD and BMD patients and with those of a control group of healthy individuals.

*Healthy control group.* The control group consisted of 22 healthy individuals without known cardiovascular disease, age-matched to the patient population, whose ECGs were used only for the ΣP/ΣQRS comparison. Detailed demographic and clinical data of the control subjects were not available in the study database.

### Statistical Analysis

Categorical variables are reported as number and percentage, with the denominator specified for each analysis. Continuous variables are reported as mean ± standard deviation (SD) and range, or as median and range, as appropriate. Baseline characteristics were compared across the three patient groups with the Kruskal–Wallis test for continuous variables and with the chi-square test for categorical variables. Because the individual P-wave and QRS voltage measurements were not retained in the study database, ΣP, ΣQRS and ΣP/ΣQRS are reported descriptively as group means and ranges, and no formal statistical comparison between groups was performed. A *p*-value < 0.05 was considered statistically significant. Analyses were performed using Python (version 3.13) with the SciPy library (version 1.18).

## 3. Results

### 3.1. Study Population

The final population consisted of 74 patients (28 DMD, 24 BMD, 22 DM1), with a total of 131 ECGs; baseline characteristics are reported in Table 1. All DMD and BMD patients were male, whereas the DM1 group included 10 females (45.5%). Age at the first ECG differed significantly between groups (*p* < 0.001), being lowest in DMD and highest in DM1 populations. Echocardiographic data were available for 50 patients: an LVEF < 45% was recorded in at least one examination in 7 of 18 DMD (38.9%) and 6 of 19 BMD patients (31.6%), with values as low as 20%, whereas all 13 DM1 patients with available echocardiography had a normal LVEF (*p* = 0.040); one additional DMD patient had a diagnosis of dilated cardiomyopathy. Three BMD patients (12.5%) and one DM1 patient (4.5%) had a permanent pacemaker, and atrial fibrillation/flutter was present in one BMD (4.2%) and four DM1 patients (18.2%).

### 3.2. Duchenne Muscular Dystrophy

**Figure 1:**
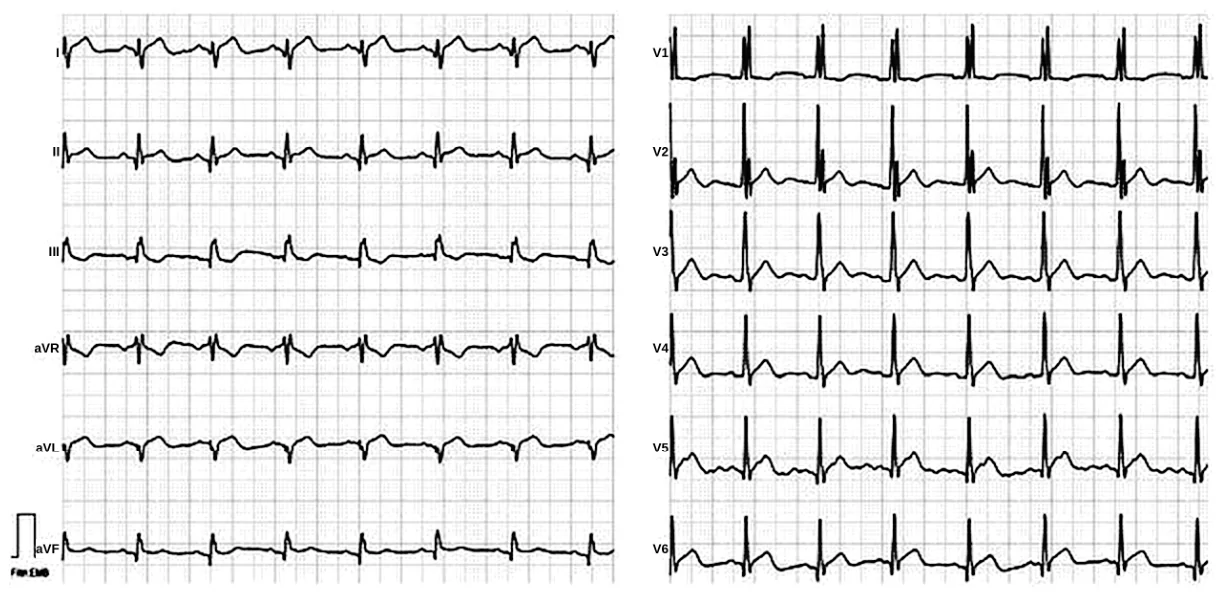
ECG of a 22-year-old patient with Duchenne Muscular Dystrophy. The tracing shows sinus rhythm at a rate of 85 bpm, normal PR interval, incomplete RBBB, with right axis deviation of the QRS. Note the tall R waves in V1 and V2 and the deep Q waves both in the inferior and in lateral leads. ST-segment elevation is also present from V3 to V6.

In the DMD group (*n* = 28), 3 patients (10.7%) showed P-wave morphology consistent with left atrial enlargement (LAE) and 5 (17.9%) showed signs of right atrial enlargement (RAE). No patient had PR prolongation, whereas a short PR interval (0.08 s) was recorded in 2 patients (7.1%). Conduction disturbances were common: 7 patients (25.0%) had incomplete RBBB and another 7 (25.0%) complete RBBB; 3 (10.7%) had left anterior fascicular block (LAFB), associated with incomplete RBBB in two of them, and 1 (3.6%) had left posterior fascicular block (LPFB) associated with incomplete RBBB. Complete left bundle branch block (LBBB) was found in 2 patients (7.1%), one of whom had incomplete LBBB on a previous ECG. A tall R wave in V1 was present in 7 patients (25.0%). Prominent Q waves were a hallmark finding and were observed in 16 of the 28 patients (57.1%): in the inferior leads in 12 (42.9%) and in the lateral leads in 10 (35.7%). Negative T waves in the lateral leads were seen in 11 patients (39.3%), and U waves were present in 7 (25.0%). An early repolarization pattern was observed in 5 patients (17.9%), and ST-segment elevation in the right and mid precordial leads in 6 (21.4%). A prolonged QT interval was reported in one patient (3.6%), who had complete RBBB. The data are summarized in Table 2. Figure 1 shows the ECG of a patient with DMD.

### 3.3. Becker Muscular Dystrophy (BMD)

**Figure 2:**
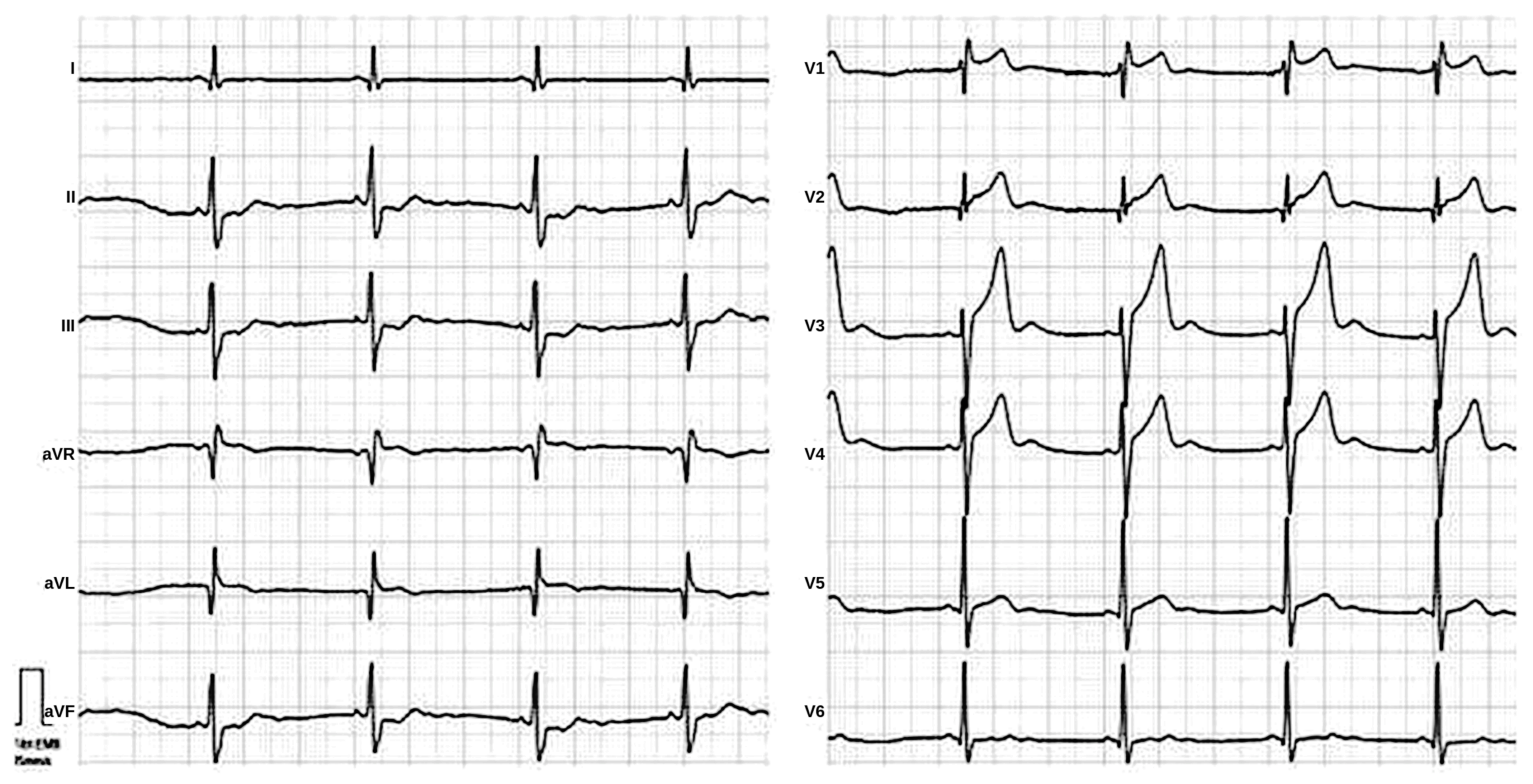
ECG of a 31-year-old patient with Becker Muscular Dystrophy. The tracing shows sinus bradycardia (52 bpm), a PR interval at the lower limit of normal, and right intraventricular conduction delay (incomplete RBBB) with an indeterminate QRS axis in the frontal plane. J-point elevation in V1–V4, aVR, and aVL, a descending ST segment in the inferior leads, and U waves from V1 to V5 are also present.

In the BMD group (*n* = 24), 3 patients (12.5%) had a permanent pacemaker implanted for third-degree AV block, and 1 patient (4.2%) had atrial fibrillation/flutter. Among the 21 patients with at least one ECG in sinus rhythm, 5 (23.8%) had P-wave morphology indicative of LAE and 1 (4.8%) of RAE, and a prolonged PR interval (0.22 s) was found in 1 patient (4.8%). Among the 22 patients with at least one non-paced ECG, intraventricular conduction abnormalities were frequent: incomplete RBBB was observed in 6 (27.3%) and complete RBBB in 3 (13.6%), one patient progressing from incomplete to complete RBBB during follow-up; complete LBBB was found in 3 (13.6%) and incomplete LBBB in 2 (9.1%). LAFB was present in 3 patients (13.6%); in one it was associated with incomplete RBBB and in another with complete RBBB. Early repolarization was present in 4 patients (18.2%). Negative T waves in the lateral leads were seen in 4 patients (18.2%), and U waves in 6 (27.3%). No QT prolongation was reported. The data are reported in Table 3. Figure 2 shows the ECG of a patient with BMD.

### 3.4. Myotonic Dystrophy Type 1 (DM1)

**Figure 3:**
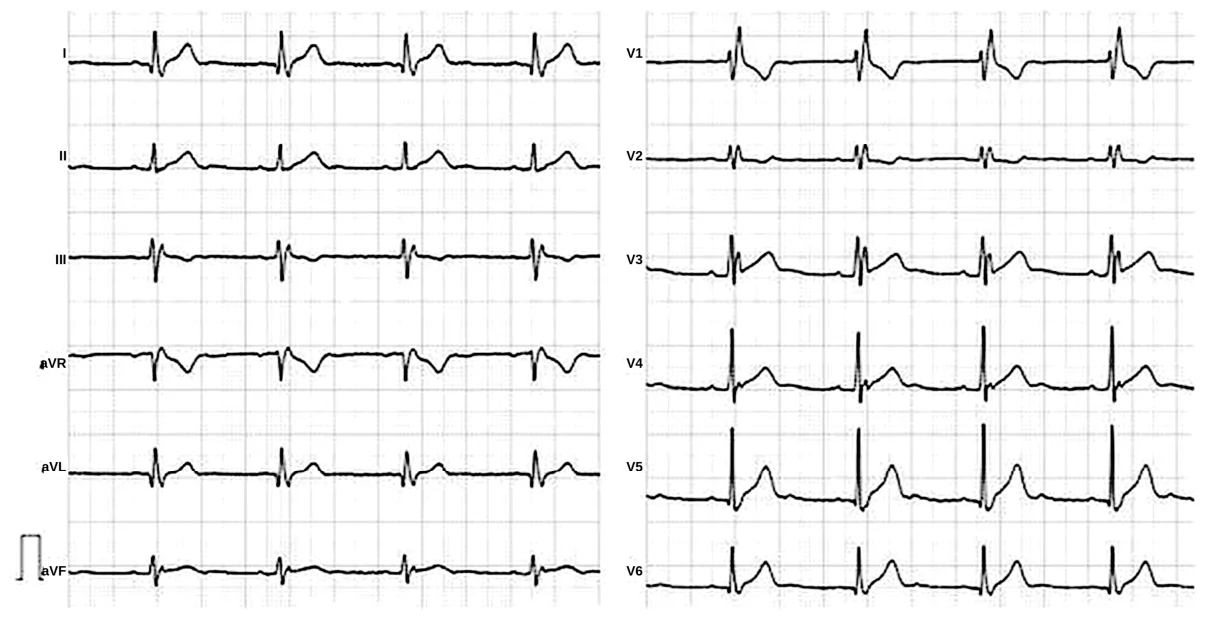
ECG of a 38-year-old male patient with Myotonic Dystrophy Type 1 (Steinert’s disease). The tracing shows sinus bradycardia 52 bpm, low P-wave voltages, normal PR interval, and a complete RBBB.

In the DM1 group (*n* = 22), 4 patients (18.2%) had atrial fibrillation (permanent in 3 and paroxysmal in 1), and 1 patient (4.5%) had a permanent pacemaker implanted for AV block. Among the 18 patients with at least one ECG in sinus rhythm, 5 (27.8%) met ECG criteria for LAE and 7 (38.9%) had a prolonged PR interval (>200 ms; range 0.22–0.30 s). Among the 21 patients with at least one non-paced ECG, complete RBBB was found in 2 (9.5%), incomplete RBBB in 2 (9.5%), complete LBBB in 3 (14.3%), incomplete LBBB in 2 (9.5%) and LAFB in 2 (9.5%). Early repolarization was remarkably common, being present in 9 patients (42.9%). T-wave morphology was generally normal, U waves were seen in 2 patients (9.5%), and no QT prolongation was reported. The data are reported in Table 4. Figure 3 shows the ECG of a patient with DM1.

The most striking finding in the DM1 group was a relatively low P-wave voltage. In the 18 DM1 patients in sinus rhythm, the mean ΣP was 0.74 mV (range 0.35–1.05 mV), the mean ΣQRS was 13.83 mV (range 7.4–24.4 mV) and the mean ΣP/ΣQRS ratio was 0.05. Mean ΣP and ΣP/ΣQRS values were lower in DM1 than in DMD (1.08 mV and 0.08, respectively), BMD (1.04 mV and 0.08) and healthy controls (1.2 mV and 0.10), whereas the mean ΣQRS was similar in DM1, DMD and BMD (13.83, 14.02 and 13.48 mV, respectively) and lower in controls (11.04 mV). The ranges of the groups partly overlapped. Because the individual voltage measurements were not retained in the study database, these data are reported descriptively and no formal statistical comparison was performed (Table 5).

## 4. Discussion

This study evaluated primary electrocardiographic alterations in a cohort of patients with DMD, BMD, and DM1, in the absence of clinically manifest heart failure. Our findings are largely consistent with the existing literature but also reveal novel elements that may offer new insights into the cardiac pathophysiology of these diseases.

### 4.1. Deep Q Waves in Duchenne Muscular Dystrophy

Our data confirm a high prevalence of deep Q waves in DMD patients, present in 57.1% of our cohort, affecting the inferior (42.9%) and lateral (35.7%) leads. This finding is a well-established ECG marker for DMD and is thought to reflect the specific anatomical pattern of cardiac involvement. The disease process often begins with fibrosis in the inferolateral wall of the left ventricle due to the absence of dystrophin. This fibrotic replacement of viable myocardium alters the propagation of the electrical impulse, creating vectors that manifest as “pseudonecrosis” or “pseudo-infarct” Q waves. Unlike the Q waves seen in hypertrophic cardiomyopathy, which are attributed to septal hypertrophy, the Q waves in DMD arise in the absence of increased wall thickness and are directly related to the fibrotic process that disrupts normal ventricular depolarization [15,16,17,18,19]. Myocardial strain imaging and CMR were not available in our retrospective cohort; therefore, the correlation between Q waves and regional (inferolateral) fibrosis or strain abnormalities could not be assessed and should be addressed in future studies combining ECGs with speckle-tracking echocardiography and late gadolinium enhancement CMR. Of note, a reduced LVEF (<45%) was recorded in at least one echocardiogram in more than one-third of DMD patients with available data, consistent with the progressive myocardial involvement of the disease.

### 4.2. Conduction Abnormalities and Repolarization

In line with previous reports, our study found a high prevalence of RBBB in both DMD (50% total) and BMD (36.4% total) patients. In DM1, both RBBB (19.0% total) and LBBB (23.8% total) were common, along with a high rate of first-degree AV block (38.9% of patients in sinus rhythm), confirming the well-known predilection of this disease for the cardiac conduction system [14,16,20,21,22,23]. A short PR interval was observed in 2 DMD patients (7.1%), a finding previously described in this disease [16].

Contrary to some reports in the literature [24,25,26], QT prolongation was rare in our cohorts: a prolonged QT interval was reported in only one DMD patient (3.6%), who had complete RBBB, so that QRS widening may have contributed to the prolongation. As numerical QT and QRS values were not stored in our database, the JTc interval could not be calculated. This is an interesting finding, as QTc prolongation is considered a marker of arrhythmic risk and has been implicated in the sudden death of patients with NMDs. The low prevalence of this finding in our population warrants further investigation. The exact incidence of sudden cardiac death in patients with neuromuscular disease, however, is not fully known; nonetheless, it appears to be low and the role of ICD implantation is still debated. Secondary prevention ICD implantation appears appropriate in survivors of spontaneously occurring significant ventricular arrhythmias or cardiac arrest, while the role of ICD implantation in primary prevention is still controversial. It is worth noting that the observation of sudden death in pacemaker and even in ICD recipients suggests that other mechanisms may be involved, including non-cardiac causes such as pulmonary embolism [27].

### 4.3. Early Repolarization

A notable finding across all three groups was the high frequency of early repolarization (ER): 17.9% in DMD, 18.2% in BMD, and a striking 42.9% in DM1. The prevalence of ER in patients with NMDs has not been adequately investigated in the literature [24,25,26,28]. The presence and the prognostic significance of ER in the general population are debated, with some considering it a benign variant and others, particularly in specific ethnic groups or locations, linking it to an increased risk of sudden cardiac death. The elevated prevalence observed in our NMD cohorts, especially in DM1, is noteworthy. However, the prevalence of ER in the general population depends on age and sex, and the different age and sex distribution of our groups (Table 1) may have contributed to the observed differences. It is plausible that the underlying myocardial pathology—fibrosis and altered cell-to-cell electrical coupling—could manifest as an ER pattern on the surface ECG. We therefore hypothesize that, in this setting, ER might reflect an abnormal myocardial substrate rather than a benign variant. However, no follow-up or outcome data were available in our study, and this hypothesis remains speculative. Prospective studies with arrhythmic outcome data are needed to clarify the role and prognostic value of ER in patients with NMDs.

### 4.4. Comparisons Between Groups

In our study, electrocardiographic abnormalities were common in all three groups of patients with the neuromuscular diseases studied. Their incidence, however, appears to differ among the different groups. For example, the presence of prominent Q waves, both in the inferior and lateral leads, appears to predominantly characterize the DMD group. Intraventricular conduction disturbances can be found in all groups, but appear to be more severe in BMD. Atrioventricular conduction disturbances, expressed by a prolongation of the PR interval, are far more frequent in the DM1 group, where they are observed in 38.9% of patients in sinus rhythm. This group also appears to be characterized by a predisposition to the onset of atrial fibrillation, which may be related to the atrial electrical abnormalities discussed below.

### 4.5. Low P-Wave Voltage in Myotonic Dystrophy Type 1

The most novel finding of our study is the observation of reduced P-wave voltage in patients with DM1, both in absolute terms (ΣP) and relative to the QRS voltage (ΣP/ΣQRS ratio). This characteristic has not been highlighted in previous literature. We hypothesize that low P-wave voltage may be an electrical expression of impaired impulse transmission within the atrial musculature, which is known to be affected by the disease process in DM1. Altered intra-atrial conduction in DM1 (P-wave duration > 120 ms) has been previously associated with atrial fibrillation risk [29,30], with “P-wave dispersion” identified as an instrumental proof. Russo et al. reported significantly increased maximum P-wave duration and P-wave dispersion in DM1 patients, particularly in those with paroxysmal atrial fibrillation, suggesting delayed and heterogeneous intra-atrial impulse propagation [29]. Increased P-wave duration reflects slowing of atrial conduction, whereas increased P-wave dispersion is considered a marker of conduction inhomogeneity and atrial electrical remodelling, both recognized substrates for atrial fibrillation [29,31]. Our finding of reduced P-wave voltage in DM1 patients may represent an additional electrocardiographic expression of this atrial involvement. Cardiovascular magnetic resonance studies have demonstrated a high prevalence of myocardial fibrosis in DM1, even in patients without overt cardiac dysfunction or advanced conduction disease [32,33]. Although not adequately investigated in the literature, it is conceivable that similar fibrotic remodelling may involve even the atrial myocardium. Such structural alterations could reduce the amount of electrically active atrial tissue, resulting in lower electrical forces recorded on the body surface and consequently lower P-wave amplitudes. Recently, some authors have hypothesized that the low voltage of the P-wave may be related to left atrial remodelling and the inter-atrial conduction pattern. This hypothesis is supported by an observational study which demonstrated that the low amplitude of the P-wave (P-wave ≤ 0.1 mV) in lead I was an independent predictor of clinical recurrence of atrial fibrillation after radiofrequency catheter ablation in patients with paroxysmal atrial fibrillation. Authors thus suggested that electrocardiograms may be a valuable tool for predicting clinical outcomes of atrial fibrillation catheter ablation using simple P-wave parameters [34]. Fibrosis is a hallmark of arrhythmogenic structural remodeling [35,36]. Tissue fibrosis results from an accumulation of fibrillar collagen deposits, occurring most commonly as a reparative process to replace degenerating myocardial parenchyma with concomitant reactive fibrosis, which causes interstitial expansion [37,38]. Fibrosis reduces conduction velocity by impairing intermyocyte coupling. Myocardial electrical continuity is maintained by specialized proteins called connexins located in gap-junctions, which form cell-to-cell connections that maintain low-resistance intercellular coupling [36].

Atrial fibrosis causes localized regions of conduction slowing, increasing conduction heterogeneity and providing a substrate for atrial fibrillation. Conduction abnormalities represent a basis for unidirectional conduction block and macro re-entry; furthermore, there is also evidence that it can promote the onset of focal arrhythmias [39]. This hypothesis is consistent with the high prevalence of atrial fibrillation and atrial flutter reported in DM1 patients [40]. Accordingly, we hypothesize that low P-wave voltage might represent an indirect electrocardiographic marker of atrial structural remodelling and increased atrial arrhythmic susceptibility in this population. However, patients with atrial fibrillation were, by design, excluded from the P-wave analysis, no longitudinal data on incident atrial arrhythmias were available, and the voltage data could only be analyzed descriptively; therefore, a direct association between low P-wave voltage and atrial arrhythmias could not be demonstrated in our cohort. Atrial fibrosis involves multifactorial processes that result from complex interactions among neurohormonal and cellular mediators. Reparative fibrosis replaces degenerating myocardial cells, whereas coexisting reactive fibrosis causes interstitial expansion between bundles of myocytes [37,38]. It is possible to hypothesize that the mitochondrial dysfunction described in DM1 may favor apoptotic cell death, promoting both reparative and reactive fibrosis in myocardial myocytes, particularly in the atria as well as in the conduction system. Whether low P-wave voltage may also identify individuals at risk of atrial fibrillation in the general population remains to be investigated. It is possible that even in patients with conditions predisposed to the onset of atrial fibrillation, such as arterial hypertension, ischemic or valvular heart disease, storage diseases, etc., the progressive fibrotic replacement of the atrial myocardium and the resulting electrical remodeling responsible for the onset of the arrhythmia might be recognized by the presence of low-voltage P-waves on the surface ECG.

Our findings suggest that low P-wave voltage could be a simple and easily obtainable ECG feature of atrial involvement in DM1. However, given the retrospective, cross-sectional and exploratory nature of this analysis, this observation should be regarded as hypothesis-generating, and its predictive value for atrial fibrillation and flutter needs to be tested in prospective longitudinal studies. The association of this finding with DM1, compared to the other NMDs studied, may suggest that the neuromuscular alterations in DM1 may have a particular tropism for atrial tissue. This could explain the high frequency of atrial flutter and atrial fibrillation observed in this specific patient population. If confirmed in larger prospective studies, this finding could contribute to risk stratification for atrial arrhythmias in DM1 patients.

### 4.6. QT Interval

Contrary to what was reported in some previous studies, a prolonged QT interval was found in only one patient (a DMD patient with complete RBBB) across the three NMD groups we studied. This finding needs to be validated in subsequent studies. If confirmed, it may be clinically relevant, as it may allow these patients to be considered for the potential use of neurological and psychotropic medications that might otherwise be considered contraindicated in the presence of QT interval prolongation.

### 4.7. Study Limitations

This study has several limitations that should be acknowledged. First, the retrospective design and the relatively small sample size of each neuromuscular disease group may limit the statistical power and generalizability of our findings, and did not allow multivariable adjustment. Second, as a single-center study, the results could be influenced by local referral patterns and specific management protocols, which may not be representative of the broader patient population. Third, ECG findings were retrieved from clinical reports in a categorical form: numerical values of intervals (including QTc and JTc) and of the individual P-wave and QRS voltages were not retained in the study database, which prevented formal statistical comparison of the voltage data; interobserver agreement was not formally assessed. Fourth, continuous ECG monitoring (Holter recordings) was not available in all patients, which prevented an accurate assessment of the incidence of rhythm disorders. Fifth, the groups differed in age and sex distribution and in the degree of left ventricular dysfunction (Table 1), which may influence ECG parameters such as QRS voltage and the prevalence of early repolarization; moreover, disease duration, genotype, comorbidities, respiratory function and non-cardiovascular therapies were not available, and the characteristics of the control group could not be reported in detail. Sixth, the ΣP analysis was exploratory and post hoc, as it was conceived after the observation of low-amplitude P waves in DM1 patients; patients with atrial fibrillation were excluded from this analysis and no longitudinal data on incident atrial arrhythmias were available, so low P-wave voltage cannot be considered a validated predictor of atrial arrhythmias. Seventh, myocardial strain imaging and CMR were not available, precluding the correlation of ECG findings with myocardial fibrosis. Finally, the cross-sectional nature of the analysis does not allow a causal link to be established between specific ECG abnormalities and long-term clinical outcomes, such as arrhythmic events or mortality; in particular, the prognostic significance of early repolarization and low P-wave voltage in NMDs remains unknown. Longitudinal studies with larger, multi-center cohorts and quantitative ECG measurements are necessary to confirm these observations.

## 5. Conclusions

We speculate that, as a result of the different genetic alterations that characterize these three distinct neuromuscular diseases, significant damage occurs at the cellular and interstitial levels, causing reparative and reactive fibrotic replacement phenomena in the myocardial tissue. This phenomenon may be common across the various forms of NMD studied; however, each of them appears to have a specific cardiac structure predisposed to the phenomenon. For example, in DMD, the phenomenon particularly affects structures such as the inferolateral wall of the left ventricle (deep Q waves), while in DM1, the main “target” structures may be the atria and atrioventricular node.

This study confirms several well-known electrocardiographic abnormalities in patients with Duchenne, Becker, and Myotonic Dystrophy Type 1, while also providing new insights.

The data reinforce the classic ECG patterns associated with each disease, such as deep Q waves in DMD and conduction system disease in DM1. Furthermore, our research has highlighted two key observations that warrant further investigation: the high prevalence of early repolarization across all three NMDs, and the novel finding of reduced P-wave voltage in patients with DM1. This latter, hypothesis-generating finding might reflect atrial myopathy in this population; whether it predicts atrial arrhythmias requires prospective validation.

Overall, our results underscore the indispensable value of routine ECG monitoring in the comprehensive management of patients with neuromuscular diseases, as it can reveal early signs of cardiac compromise and help guide therapeutic strategies to improve patient outcomes and quality of life.

## Figures and Tables

**Table 1:** Baseline characteristics of the study population.

Characteristic	DMD (*n* = 28)	BMD (*n* = 24)	DM1 (*n* = 22)	*p*-Value
Age at first ECG, years, mean ± SD (range)	23.0 ± 6.4 (10–37)	39.2 ± 17.2 (16–79)	43.2 ± 10.8 (26–76)	<0.001
Male sex, *n* (%)	28 (100)	24 (100)	12 (54.5)	<0.001
Follow-up, years, mean ± SD	3.8 ± 3.1	4.2 ± 3.1	2.4 ± 2.1	0.067
ECGs per patient, median (range)	2 (1–2)	2 (1–2)	2 (1–3)	0.631
Echocardiography available, *n*	18	19	13	—
LVEF < 45% in ≥1 examination, *n*/*N* (%)	7/18 (38.9)	6/19 (31.6)	0/13 (0)	0.040
Left ventricular dilation, *n*/*N* (%)	5/18 (27.8)	6/19 (31.6)	0/13 (0)	0.081
Left atrial dilation, *n*/*N* (%)	0/18 (0)	2/19 (10.5)	1/13 (7.7)	0.386
Permanent pacemaker, *n* (%)	0 (0)	3 (12.5)	1 (4.5)	0.136
Atrial fibrillation/flutter, *n* (%)	0 (0)	1 (4.2)	4 (18.2)	0.033

Continuous variables were compared with the Kruskal–Wallis test and categorical variables with the chi-square test. BMD, Becker Muscular Dystrophy; DM1, Myotonic Dystrophy Type 1; DMD, Duchenne Muscular Dystrophy; LVEF, left ventricular ejection fraction.

**Table 2:** ECG abnormalities in Duchenne Muscular Dystrophy (28 patients).

ECG Abnormality	*n*/*N*	Percentage
Left atrial enlargement (LAE)	3/28	10.7%
Right atrial enlargement (RAE)	5/28	17.9%
PR prolongation (>200 ms)	0/28	0.0%
Short PR interval (<120 ms)	2/28	7.1%
Incomplete RBBB	7/28	25.0%
Complete RBBB	7/28	25.0%
Incomplete LBBB	1/28	3.6%
Complete LBBB	2/28	7.1%
LAFB	3/28	10.7%
LPFB	1/28	3.6%
Tall R in V1	7/28	25.0%
Deep Q waves (any)	16/28	57.1%
Deep Q waves, inferior leads	12/28	42.9%
Deep Q waves, lateral leads	10/28	35.7%
Negative T waves (lateral leads)	11/28	39.3%
U waves	7/28	25.0%
Early repolarization (ER)	5/28	17.9%
ST elevation, right/mid precordial leads	6/28	21.4%
Prolonged QT interval	1/28	3.6%

**Table 3:** ECG abnormalities in Becker Muscular Dystrophy (*n* = 24). Percentages are calculated on the number of patients evaluable for each finding (*n*/*N*).

ECG Abnormality	*n*/*N*	Percentage
Permanent pacemaker	3/24	12.5%
Atrial fibrillation/flutter	1/24	4.2%
Left atrial enlargement (LAE)	5/21	23.8%
Right atrial enlargement (RAE)	1/21	4.8%
PR prolongation (>200 ms)	1/21	4.8%
Incomplete RBBB	6/22	27.3%
Complete RBBB	3/22	13.6%
Incomplete LBBB	2/22	9.1%
Complete LBBB	3/22	13.6%
LAFB	3/22	13.6%
Negative T waves (lateral leads)	4/22	18.2%
U waves	6/22	27.3%
Early repolarization (ER)	4/22	18.2%
Prolonged QT interval	0/22	0.0%

**Table 4:** ECG abnormalities in Myotonic Dystrophy Type 1 (*n* = 22). Percentages are calculated on the number of patients evaluable for each finding (*n*/*N*).

ECG Abnormality	*n*/*N*	Percentage
Atrial fibrillation, permanent	3/22	13.6%
Atrial fibrillation, paroxysmal	1/22	4.5%
Permanent pacemaker	1/22	4.5%
Left atrial enlargement (LAE)	5/18	27.8%
PR prolongation (>200 ms)	7/18	38.9%
Incomplete RBBB	2/21	9.5%
Complete RBBB	2/21	9.5%
Incomplete LBBB	2/21	9.5%
Complete LBBB	3/21	14.3%
LAFB	2/21	9.5%
U waves	2/21	9.5%
Early repolarization (ER)	9/21	42.9%
Prolonged QT interval	0/21	0.0%

**Table 5:** P-wave and QRS voltage sums in subjects in sinus rhythm. Values are mean (range).

Parameter	DM1 (*n* = 18)	DMD (*n* = 28)	BMD (*n* = 21)	Healthy Controls (*n* = 22)
ΣP (mV)	0.74 (0.35–1.05)	1.08 (0.58–1.7)	1.04 (0.5–1.6)	1.2 (0.8–1.9)
ΣQRS (mV)	13.83 (7.4–24.4)	14.02 (8.3–26.5)	13.48 (7.5–24.5)	11.04 (7.5–19.8)
ΣP/ΣQRS	0.05	0.08	0.08	0.10

## Data Availability

The de-identified individual data supporting the reported results are provided in Supplementary Table S1; further data are available from the corresponding author upon reasonable request.

## Supplementary Materials

The following supporting information can be downloaded at https://www.mdpi.com/article/10.3390/diagnostics16213582/s1, Table S1: De-identified individual patient data (disease group, sex, age, follow-up, rhythm status, pacemaker, LVEF, chamber dilation and ECG findings).

## Abbreviations

The following abbreviations are used in this manuscript:

AF	atrial fibrillation
AV	atrioventricular
BMD	Becker Muscular Dystrophy
CMR	cardiovascular magnetic resonance
DMD	Duchenne Muscular Dystrophy
DM1	Myotonic Dystrophy Type 1
ECG	electrocardiogram
ER	early repolarization
HF	heart failure
ICD	implantable cardioverter-defibrillator
JTc	corrected JT interval
LAE	left atrial enlargement
LAFB	left anterior fascicular block
LBBB	left bundle branch block
LPFB	left posterior fascicular block
LVEF	left ventricular ejection fraction
mV	millivolt
NMDs	neuromuscular diseases
NS	not significant
PR	PR interval
QTc	corrected QT interval
RAE	right atrial enlargement
RBBB	right bundle branch block
RNA	ribonucleic acid
*SCN5A*	sodium voltage-gated channel alpha subunit 5 gene
SD	standard deviation
ST	ST segment
ΣP	sum of absolute P-wave voltages across the 12 ECG leads
ΣQRS	sum of absolute QRS voltages across the 12 ECG leads
ΣP/ΣQRS	ratio of the P-wave voltage sum to the QRS voltage sum
